# Chronic *Serratia marcescens* sternal infection presenting 13 years after coronary artery surgery

**DOI:** 10.1016/j.ijscr.2019.08.007

**Published:** 2019-08-17

**Authors:** Ashley Chinn, Michael Knabel, James R. Sanger, Paul S. Pagel, G. Hossein Almassi

**Affiliations:** a Cardiothoracic Surgery, Medical College of Wisconsin, Milwaukee, WI, United States; b Department of Surgery, Clement J. Zablocki Veterans Affairs Medical Center, Milwaukee, WI, United States; c Department of Plastic Surgery, Medical College of Wisconsin, Milwaukee, WI, United States; d Department of Anesthesiology, Clement J. Zablocki Veterans Affairs Medical Center, Milwaukee, WI, United States

**Keywords:** Sternal infection, Mediastinitis, Coronary artery disease, CABG, *Serratia mercescens*

## Abstract

**Introduction:**

*Serratia marcescens* is a facultative anaerobic bacillus that very rarely causes sternal infections. We describe a sternal abscess resulting from chronic *S. marcescens* infection that presented 13 years after coronary artery bypass graft surgery (CABG).

**Presentation of case:**

A 71-year-old diabetic man presented 13 years after CABG with a new distal sternal “mass” that intermittently drained purulent fluid. He was treated with oral antibiotics, but the symptoms persisted. Exploration revealed an abscess extending to the sternal body. A non-absorbable braided suture and a sternal wire were removed, but a sinus tract remained despite further antibiotics and conservative care. Subsequent computed tomography and bone scintigraphy revealed a substernal soft tissue density with bone involvement. An abscess cavity was excised from the substernal anterior mediastinum. Another non-absorbable braided suture was removed. Cultures grew carbapenem-resistant *S. marcescens*.

**Discussion:**

Nosocomial or hospital-associated clusters of *S. marcescens* infection are known, but isolated infections seldom occur. *S. marcescens* infections in cardiac surgery patients are unusual. Only a single report described a chronic sternal infection resulting from *S. marcescens* that was identified 15 years after an initial episode caused by the same organism in a heart transplant recipient who was immunocompromised. Diabetes and non-absorbable braided sutures placed for hemostasis at the wire sites were probably contributing factors to our patient’s chronic infection.

**Conclusion:**

This report described the presentation and treatment of a chronic *S. marcescens* sternal abscess that occurred 13 years after CABG. Chronic sternal infections due to this organism in cardiac surgery patients are exceeding rare.

## Highlights

• *Serratia marcescens* is gram-negative facultative anaerobic bacillus.
• *S. marcescens* is responsible for a small percentage of hospital-acquired infections.
• *S. marcescens* infection usually occurs in outbreaks.
• This case describes a rare chronic *S. marcescens* sternal infection.

## 1 Introduction

Sternal infections are uncommon after coronary artery bypass graft surgery (CABG) [1]. *Serratia marcescens* is gram-negative facultative anaerobic bacillus in the *Enterobacteriaceae* family that is responsible for a small percentage of hospital-acquired infections (e.g., urinary tract, wound, catheter-related) [2]. These pathogens very rarely cause sternal infections [3,4], in contrast to those resulting from more common organisms such as coagulase negative *Staphylococcus*, *S. aureus*, and other gram-negative bacteria [5]. We describe a patient who presented with a localized sternal abscess resulting from chronic *Serratia marcescens* infection 13 years after CABG. Written informed consent was obtained from the patient for publication of this case report and its accompanying images. This work has been reported in line with the SCARE criteria [6].

## 2 Presentation of case

**Fig. 1:**
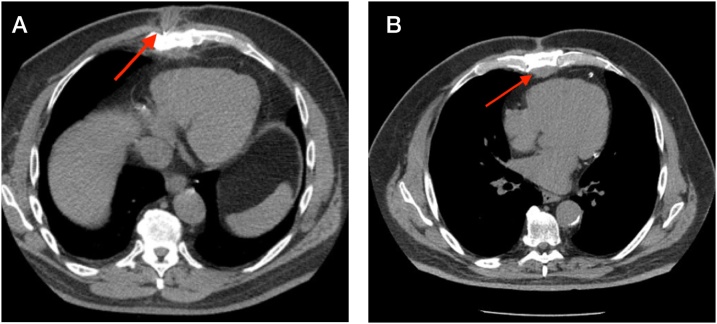
Axial thoracic computed tomography (CT) image showing soft tissue density anterior to the sternum (red arrow, panel A); subsequent CT image showing soft tissue density posterior to the sternum (red arrow, panel B).

A 71-year-old man with a history of coronary artery disease, poorly-controlled diabetes mellitus, and prostate cancer presented to our institution for evaluation of recurrent sternal drainage. The patient underwent CABG at another hospital 13 years before the current admission; the authors did not have access to the medical records from this outside hospital. His postoperative recovery was complicated by a superficial sternal infection, which was successfully treated with iodoform packing and a course of oral antibiotics. The patient’s subsequent clinical course was unremarkable from an infectious disease perspective, but he did require several percutaneous coronary interventions during the intervening years because of additional symptomatic coronary stenoses. The patient stated that he had visited the emergency department three months earlier because of a new “mass” located in the distal sternum that intermittently drained purulent fluid. The patient was treated with oral sulfamethoxazide-trimethoprim, but his symptoms persisted, prompting his return to the hospital. He denied trauma, fever, chills, and recent illness. The physical examination revealed a small (1 cm), mildly tender, erythematous wound at the inferior aspect of the healed sternotomy scar. A scant amount of creamy yellow drainage was expressed from the opening, but a tunnel could not be identified with a probe. The patient was managed conservatively with wound care, but he continued to have purulent drainage from the site. Computed tomography revealed a small density that was consistent with a soft-tissue abscess superficial to the lower sternum (Fig. 1A).

Exploration of the wound in the operating room indicated that the abscess extended down to the body of the sternum. A non-absorbable braided suture was identified that was wrapped around a sternal wire at the wound base. The suture and sternal wire were removed. The surrounding soft tissue and sternal bone were extensively debrided, but retrosternal communication of the infection was absent. After irrigation with bacitracin solution, the wound was closed primarily with absorbable suture. The patient tolerated the procedure well and was treated with oral sulfamethoxazide-trimethoprim as an outpatient. Culture of tissue samples from the wound, including the suture, were positive for multidrug resistant *Serratia marcescens*, but the organism demonstrated sensitivity to sulfamethoxazide-trimethoprim. This medication was continued as a result. The patient’s wound required reopening during a subsequent clinic appointment, which was treated with packing and a vacuum dressing.

**Fig. 2:**
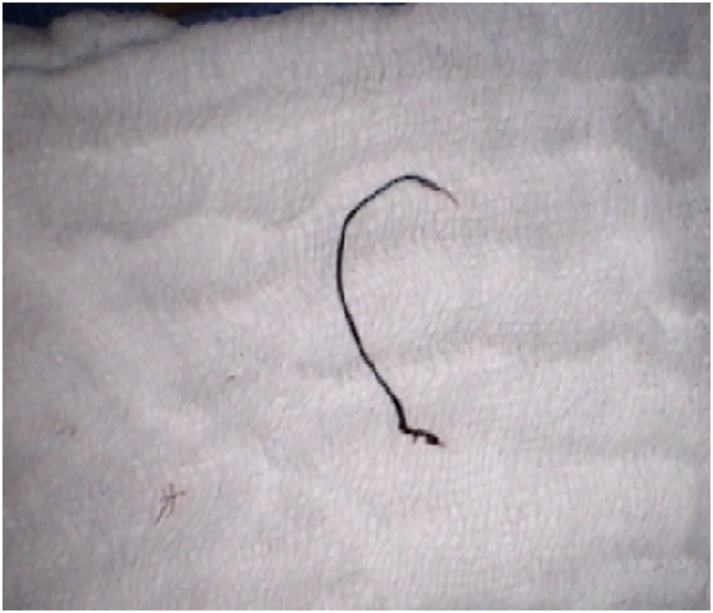
Braided non-absorbable suture removed from the sternum; the suture grew multidrug resistant *Serratia marcescens.*

**Fig. 3:**
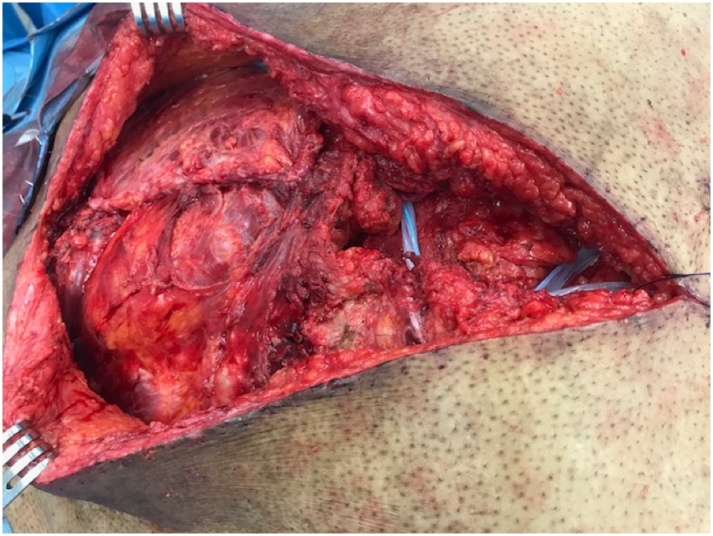
Intraoperative photograph showing sternal closure with bilateral pectoralis muscle flaps.

The patient’s wound gradually healed during the ensuing three months, but a sinus tract persisted that intermittently drained purulent fluid. A new computed tomography scan now revealed a localized substernal soft tissue density (Fig. 1B) and bone scintigraphy demonstrated radioisotope enhancement in the sternum at this site. The patient was taken back to the operating room for additional exploration. A repeat sternotomy was performed. A thick-walled abscess cavity was excised from the substernal anterior mediastinum. Another non-absorbable braided suture was also identified immediately superior to the location of the previously excised suture (Fig. 2). The abscess was excised and the suture was removed. Sternal bone cultures subsequently grew carbapenem-resistant *Serratia marcescens*. After a sternecotomy was completed, the defect was closed with bilateral pectoralis muscle flaps (Fig. 3). The patient was treated with a six-week course of intravenous piperacillin and tazobactam. He made an uneventful recovery.

## 3 Discussion

Environmental sources are usually responsible for *Serratia* infections [2]. The most virulent human pathogen among these organisms is *S. marcescens*, which may be acquired through contact with stagnant water, soil, or plants [2]. Nosocomial or hospital-associated clusters of *S. marcescens* infection have also been described, but isolated infections rarely occur in individual patients independent of an outbreak, such as was observed in our patient. Many hospital-based outbreaks of *S. marcescens* were reported resulting from point sources (e.g., tap water, disinfectant solutions, hand lotion, soap), medications (e.g., intravenous fluids, blood products, the intravenous anesthetic propofol), and inadequate hand hygiene among health care workers [[7], [8], [9]]. Postoperative skin, soft tissue, or fascia infections with or without necrosis have also been described, but such infections continue to be unusual [7,9]. When they do occur, *S. marcescens* infections often demonstrate clinical features that are similar to those observed with infections caused by other, more commonly encountered organisms [2].

Reports describing *S. marcescens* infections in cardiac surgery patients are sparse and those involving the sternum are exceeding rare. In 1987, Wilhemi et al. first described an epidemic outbreak of *S. marcescens* surgical wound infections in ten postoperative cardiac surgical patients in Spain, half of whom had sternal osteomyelitis [4]. The organism was eventually isolated from the equipment and hands of the barbers who were responsible for shaving the patients before surgery. Another series of acute *S. marcescens* infections in 14 adult cardiac surgery patients, one of which involved the sternum, was traced to six reusable 12-lead electrocardiogram bulbs [10]. Several acute *S. marcescens* infections that occurred in cardiac patients were linked to contaminated bottles containing a solution used to disinfect cardiopulmonary bypass equipment that had been improperly cleaned [8]. Failure to comply with established infection prevention protocols caused another outbreak of *S. marcescens* in a cardiothoracic intensive care unit [11]. *S. marcescens* was responsible for one of 12 acute blood stream infections after median sternotomy in 192 patients undergoing cardiac surgery at a major children’s hospital [12]. To our knowledge, only a single case report previously described a chronic sternal infection resulting from *S. marcescens* that was identified 15 years after an initial episode caused by the same organism in a heart transplant recipient treated with immunosuppressant medications [3]. The authors speculated that bacterial persistence and reemergence may have occurred as a consequence of limited antibiotic penetration into the site of infection, phenotypic adaptation by the organism, and the possible existence of dormant bacteria in a patient who was chronically immunosuppressed [3,[13], [14], [15]]. Similar mechanisms may have been responsible for the chronic *S. marcescens* infection in our patient with poorly-controlled diabetes, a known risk factor for postoperative infection, including those caused by *S. marcescens*, after cardiac surgery [11,16,17]. However, it was also apparent that non-absorbable braided sutures placed around sternal wires (presumably for hemostasis at the wire sites) were a contributing factor to our patient’s chronic infection.

## 4 Conclusion

This report describes the presentation and treatment of a chronic *Serratia marcescens* sternal abscess that occurred 13 years after CABG. Chronic sternal infections due to this organism in cardiac surgery patients are exceeding rare.

## Sources of funding

There are no sources of funding.

## Ethical approval

The case report is not a research study. This section is not applicable.

## Consent

Written informed consent was obtained from the patient for publication of this case report and accompanying images. A copy of the written consent is available for review by the Editor-in-Chief of this journal on request.

## Author contributions

Ashley Chinn BS: Collected relevant medical records, conducted the literature search, and wrote the first draft of the manuscript; this author approves the final version of the manuscript to be submitted.

Michael Knabel BS: Helped obtain relevant medical records, provided additional literature; writing and critical editing the manuscript through several drafts; this author approves the final version of the manuscript to be submitted.

James R. Sanger MD: Performed the reconstructive surgery; writing and critical editing the manuscript through several drafts; this author approves the final version of the manuscript to be submitted.

Paul S. Pagel MD PhD: Provided anesthesia for several of the patient’s operations; writing and critical editing the manuscript through several drafts; this author approves the final version of the manuscript to be submitted.

G. Hossein Almassi MD: Performed sternal debridement procedures; writing and critical editing the manuscript through several drafts; this author approves the final version of the manuscript to be submitted.

## Registration of research studies

Not applicable.

## Guarantor

G. Hossein Almassi MD accepts full responsibility for the work and controlled the decision to publish.

## Provenance and peer review

Not commissioned, externally peer-reviewed.

## Declaration of Competing Interest

The authors have no conflicts of interest.
